# Association between follicular fluid bacteria with inflammatory markers of the complete blood count and the outcomes of assisted reproductive technology in women with endometriosis: A case–control study

**DOI:** 10.1002/hsr2.1874

**Published:** 2024-02-08

**Authors:** Roya Kabodmehri, Seyedeh Hajar Sharami, Sedigheh Tavakoli, Yalda Donyaei‐Mobarrez, Ziba Zahiri Sorouri, Nasrin Ghanami Gashti, Mohammad hadi Bahadori, Forozan Milani

**Affiliations:** ^1^ Department of Obstetrics & Gynecology, School of Medicine, Reproductive Health Research Center, Al‐Zahra Hospital Guilan University of Medical Sciences Rasht Iran; ^2^ Department of Midwifery, School of Nursing and Midwifery Guilan University of Medical Sciences Rasht Iran; ^3^ Mehr Fertility Research Center Guilan University of Medical Sciences Rasht Iran; ^4^ Department of Obstetrics & Gynecology, School of Medicine, Reproductive Biology/Reproductive Health Research Center, Al‐Zahra Hospital Guilan University of Medical Sciences Rasht Iran; ^5^ Cellular and Molecular Research Center, School of Medicine Guilan University of Medical Sciences Rasht Iran

**Keywords:** bacteria, endometriosis, follicular fluid, infertility

## Abstract

**Background and Aims:**

Endometriosis is a common reason for infertility and poor outcomes of assisted reproductive technology (ART). Inflammation is involved in the pathogenesis of this disease. The presence of microorganisms in women with endometriosis may increase levels of inflammatory markers. The purpose of this study is to determine the relationship between the presence of bacteria in the follicular fluid with the inflammatory markers of the complete blood count (CBC) and the outcomes of in vitro fertilization (IVF) in women with endometriosis.

**Methods:**

This case–control study was conducted on 74 patients undergoing IVF, referred to Al‐Zahra Hospital in Rasht (Iran) in 2021. The patients were divided into two case groups including 37 women with endometrioma and the control group, including 37 infertile women with a male factor and normal ultrasound. In total, 74 follicular fluids were collected from the case and control groups and were cultured in the laboratory. The relationship between culture results with IVF outcomes and the levels of CBC inflammatory markers including the number of white blood cells (WBCs), lymphocytes, neutrophils, neutrophil‐to‐lymphocyte ratio (NLR), lymphocyte‐to‐monocyte ratio (LMR), and platelet‐to‐lymphocyte ratio (PLR), erythrocyte sedimentation rate (ESR), and c‐reactive protein (CRP) was analyzed.

**Results:**

There was no significant statistical difference between the frequency of bacteria present in the follicular fluid (*p* = 0.861), the mean rate of fertilization (*p* = 0.363), the frequency of CRP (*p* = 0.999), and the mean WBCs, lymphocytes, neutrophils, NLR, LMR, and PLR in the two groups. There was a significant statistical difference between the mean number of oocytes of metaphase II (*p* = 0.034) and the mean ESR (*p* = 0.018) in the two groups.

**Conclusions:**

It seems necessary to evaluate follicular fluid as a biological substance that is considered an optimal factor for predicting oocyte quality, fertilization rate, embryo quality, and the success rate of ART.

## 1 INTRODUCTION

Endometriosis is a chronic inflammatory disease characterized by the growth of endometrial tissue outside the uterine cavity and affects 10% of reproductive‐aged women.^1^, ^2^ Endometriosis may extend to the ovaries and result in the formation of an endometrioma cyst.^2^, ^3^ Approximately 17%–44% of women diagnosed with endometriosis have an endometrioma.^3^ Endometriosis is a common cause of infertility. About 25%–50% of infertile women have endometriosis, and 30%–50% of women with endometriosis are infertile.^2^

According to studies, endometriosis as an inflammatory disease is associated with dysfunction of immune cells, especially macrophages, and abnormal and excessive production and secretion of immune mediators such as cytokines, prostaglandins, and metalloproteases.^4^, ^5^ In other words, in women with endometriosis, ectopic endometrial tissue increases the number of macrophages in the peritoneal cavity by releasing cytokines and chemokines. An increase in the macrophages may also increase the inflammatory factors such as monocyte chemotactic protein‐1, tumor necrosis factor‐α, interleukin‐8 (IL‐8), and IL‐6 and result in the worsening of the inflammation.^6^, ^7^ An increase in proinflammation factors such as IL‐6 may also result in an increase of other inflammation markers such as c‐reactive protein (CRP).^8^

Regarding the endometriosis pathogenesis, which is based on the presence of chronic inflammation, the disease might affect the tubo‐ovarian function, transport of gametes, endocrine changes, changes in the capacity of the endometrium, inflammation, and destruction of the ovarian cortical, and decreasing the quality and quantity of oocytes, and result in subfertility or infertility in women.^1^, ^9^, ^10^, ^11^

Assisted reproductive technology (ART), especially in vitro fertilization (IVF)/intracytoplasmic sperm injection (ICSI), is one of the most widely used treatment strategies in infertile women with endometriosis.^3^ Follicular fluid plays an important role in steroidogenesis, follicle growth, oocyte maturation, ovulation, and oocyte transport, and is an optimal factor for predicting oocyte quality, fertilization rate, embryo quality, and IVF/ICSI success.^12^, ^13^

According to studies, although follicular fluid has antimicrobial activity, this fluid is not always sterile and may be contaminated by various microorganisms.^13^, ^14^, ^15^ Cytokines secreted by follicular fluid microorganisms may lead to exacerbation of inflammation in patients with endometriosis and have a negative effect on fertility rate and IVF/ICSI results.^6^, ^16^, ^17^, ^18^ In other words, microbial colonization of the upper genital tract is one of the independent factors that influence the success rate of ART.^19^ According to a research conducted to evaluate the effect of microbial colonization of follicular fluid on the outcomes of ART, bacterial colonization in follicular fluid was associated with a decrease in fertilization rate and viable embryos in ART cycles, especially in women with endometriosis.^17^

Endometriosis is associated with inflammation, so^4^, ^20^ the systemic inflammatory response can be associated with changes in different categories of white blood cells (WBCs).^20^ The neutrophil‐to‐lymphocyte ratio (NLR) and platelet‐to‐lymphocyte ratio (PLR) in peripheral blood are considered as simple indicators of systemic inflammatory response and disease prognosis.^20^, ^21^ According to a research that was done to assess the diagnostic value of NLR alone and combined with CA‐125 for the diagnosis of endometriosis, the mean NLR and the combined marker of NLR and CA‐125 in patients with endometriosis were significantly higher than patients without endometriosis. In this study, NLR was also able to distinguish patients with endometriosis from patients with benign ovarian tumors and healthy people.^20^

Studies suggest that prescribing broad‐spectrum antibiotics may be effective in treating human endometriosis by reducing inflammation, cell proliferation, and angiogenesis.^22^, ^23^ Vaginal antibiotics may help treat endometriosis, according to a study in animal models, but more research is needed before the results can be generalized to humans.^24^

As one of the clinical predictors of inflammation diagnosis is the complete blood count (CBC)‐based biomarkers and this evaluation is an available cost‐effective method, the purpose of this study is to determine the relationship between the presence of bacteria in follicular fluid with the inflammatory markers of the CBC and the outcomes of IVF in women with endometriosis.

## 2 MATERIALS AND METHODS

### 2.1 Study design and setting

The present research was a case–control study that was performed to determine the relationship between the presence of bacteria in follicular fluid with the inflammatory markers of the CBC and the outcomes of IVF in women with endometriosis who were referred to the infertility clinic of Al‐Zahra Hospital in Rasht (Iran) in 2021–2022.

### 2.2 Sample size, sampling, and study participants

This case–control study was conducted between November 2021 and October 2022. Participants were 74 women aged 18–40 undergoing IVF. The sample size was determined based on a confidence interval (CI) of 90% and power of 80%, and after 10% attrition, the number of participants in each group was determined to be 37. The participants were divided into case group including 37 women with endometriosis identified by ultrasound (presence of endometrioma) and the control group, including 37 infertile women with a male factor and normal ultrasound and no signs of the presence of endometriosis or other infertility‐related diseases. Both case and control groups were matched in terms of age, body mass index (BMI), and ovarian stimulation protocol. The exclusion criteria include patients who have not responded to ovarian stimulation and recent antibiotic use within the past 3 months.

The participants were included in the study by simple sampling method. IVF treatment started for both groups after informed consent.

The evaluated inflammatory markers included studying the number of WBC, lymphocytes, neutrophils, NLR, lymphocyte‐to‐monocyte ratio (LMR), PLR, erythrocyte sedimentation rate (ESR), and c‐reactive protein (CRP).

To evaluate follicular fluid and perform IVF, 2 mg of estradiol was prescribed twice a day from the 20th day of the previous cycle. On the second day of the cycle, ultrasound was performed, and if there was no follicle above 10 mm, ovarian stimulation was induced by follicle‐stimulating hormone (Cinnal‐F, CinnaGen)/human menopausal gonadotropin (PD HOMOG). The ovarian response to the stimulation was evaluated by ultrasound. The first ultrasound was performed 5–6 days after ovulation stimulation. If there were 13–14 mm follicles in the ultrasound, gonadotropin‐releasing hormone antagonist (Cetreotide, 0.25 mg; Merck) was prescribed to prevent luteinizing hormone surge. The ovarian stimulation was stopped after observing at least two 18 mm follicles in ultrasound, and 10,000 U of human chorionic gonadotropin (hCG) was injected. About 36–40 h after administration of hCG, ovarian follicles were aspirated under anesthesia and using a vaginal ultrasound guide. After IVF, the embryos were classified into four A–D grades based on quality.

In this study, the follicular fluid was aspirated under sterile conditions and sent to the laboratory to detect the presence of bacteria in follicular fluid from the largest and most available follicles (17–20 mm) in the left or right ovary. The result of the culture was the number of bacterial colonies and the type of bacteria (Gram‐positive or Gram‐negative).

### 2.3 Data analysis

The research was conducted using SPSS version 21. The normality test of continuous variables was done by the Shapiro−Wilk test. Normally distributed continuous variables were described by mean and standard deviation; nonnormally distributed continuous variables were described by median and interquartile range (IQR); categorical variables were described by count and percentage. To compare the means of two‐category variables, the independent‐samples *t*‐test was used. The Mann−Whitney *U*‐test was utilized for nonparametric comparisons, such as the mean number of oocytes, the mean rate of fertilization, and the mean inflammatory markers between the two groups. To measure the relationship between qualitative variables, the *χ* ^2^ test was used. All the above analyses were considered statistically significant with *p* < 0.05.

## 3 RESULTS

The participants in this study were 74 women between the ages of 18 and 40 who had undergone IVF, and none of them were excluded from the study. Based on the findings of this study, the mean age of women with and without endometriosis was 35.38 ± 4.79 and 35.84 ± 5.52, respectively. There was no significant statistical difference between the mean ages of the two groups (*p* = 0.566).

The mean BMI in women with and without endometriosis was 26.98 ± 2.63 and 25.67 ± 2.47, respectively. There was no significant statistical difference between the mean BMI of the two groups (*p* = 0.051).

The presence of bacteria in follicular fluid in the endometriosis and nonendometriosis groups included 21 (56.7%) and 32 (59.5%) cases, respectively, and all the bacteria were Gram‐positive in both groups. There was no significant statistical difference between the frequencies of bacteria present in the follicular fluid of the two groups (*p* = 0.861). Additional information is inserted in Table 1.

**Table 1:** Distribution of frequency of the bacteria present in follicular fluid of the two groups of women with and without endometriosis.

Variable	Groups		*p* Value
	Case	Control	
Presence of bacteria in follicular fluid			
In the right ovary	6 (16.2%)	4 (10.8%)	0.861
In the left ovary	5 (13.5%)	7 (18.9%)	
In both ovaries	10 (27.0%)	11 (29.7%)	
Absence	16 (43.3%)	15 (43.3%)	

The mean number of metaphase II (M II) oocytes in women with and without endometriosis was 4.22 ± 3.58 and 6.51 ± 4.79, respectively. There was a significant statistical difference between the mean numbers of M II oocytes of the two groups (*p* = 0.034).

The mean fertilization rate of women with and without endometriosis was 61.73 ± 34.22 and 68.35 ± 34.56. There was no significant statistical difference between the mean fertilization rates of the two groups (*p* = 0.363).

The distribution of the mean quality of the embryo in grades A–D in both groups of women with or without endometriosis is included in Table 2. There was no significant statistical difference between the mean embryo qualities in grades A, B, C, and D in the two groups.

**Table 2:** Distribution of the mean embryo quality in the two groups of women with and without endometriosis.

	Embryo quality				*p* Value
	Mean ± SD	Median (IQR)	Minimum	Maximum	
Grade A					
Case	69.40 ± 39.05	100 (100, 33.3)	12.5	100	0.272
Control	41.16 ± 34.55	33.3 (70, 16.3)	14.3	100	
Grade B					
Case	57.94 ± 34.02	50 (100, 26.7)	12.5	100	0.399
Control	44.54 ± 25.71	33.3 (58.4, 26.2)	20	100	
Grade C					
Case	65.68 ± 30.75	71.4 (100, 33.3)	16.6	100	0.311
Control	74.06 ± 30.87	88.9 (100, 50)	18.2	100	
Grade D					
Case	24.26 ± 16.84	20 (37.5, 11.3)	7.7	53.8	0.057
Control	46.37 ± 33.46	33.3 (87.5, 21.3)	11.1	100	

Abbreviation: IQR, interquartile range.

The distribution of mean blood inflammatory markers in the two groups of women with and without endometriosis is inserted in Table 3. There was no significant statistical difference between the mean WBC, lymphocytes, neutrophils, NLR, LMR, and PLR in the two groups.

**Table 3:** Distribution of mean blood inflammatory markers in the two groups of women with and without endometriosis.

	Embryo quality				
	Mean ± SD	Median (IQR)	Minimum	Maximum	*p* Value
LMR					
Case	6.42 ± 3.21	5.6 (8.8, 3.7)	2.5	15	0.974
Control	6.78 ± 4.03	5.3 (10.2, 3.7)	2.5	20	
PLR					
Case	9.50 ± 4.37	9.3 (10.5, 6.5)	4.6	25	0.661
Control	8.80 ± 2.61	8.7 (10.4, 7.4)	4.7	18.1	
NLR					
Case	2.46 ± 1.11	2.3 (2.8, 1.8)	1.1	7	0.294
Control	2.25 ± 0.92	2.1 (2.6, 1.7)	1.2	5.7	
Lymphocyte					
Case	28.69 ± 6.87	28 (33.4, 24.5)	12	43	0.372
Control	29.84 ± 6.76	31 (33.5, 25.5)	14	41	
Neutrophil					
Case	64.56 ± 8.74	65 (71, 58)	45	84	0.143
Control	61.67 ± 7.78	62 (68, 56.5)	48	80	
WBC					
Case	7.78 ± 2.09	7.3 (8.2, 6.5)	4.7	13	0.808
Control	7.89 ± 2.11	7.2 (9.5, 6.3)	4.2	12.9	

Abbreviations: IQR, interquartile range; LMR, lymphocyte‐to‐monocyte ratio; NLR, neutrophil‐to‐lymphocyte ratio; PLR, platelet‐to‐lymphocyte ratio; WBC, white blood cell.

The CRP frequency in the groups of women with and without endometriosis was two persons (5.4%) and one person (2.7%) respectively. There was no significant statistical difference between the CRP frequencies of the two groups (*p* = 0.999).

The mean ESR in women with and without endometriosis was 20.48 ± 14.05 and 15.81 ± 10.91, respectively. There was a significant statistical difference between the ESR frequencies of the two groups (*p* = 0.018).

## 4 DISCUSSION

The present study was conducted to investigate the relationship between the presence of bacteria in follicular fluid with the inflammatory markers of the CBC and the outcomes of IVF in women with endometriosis. In line with the results of this study, based on a study that was performed to compare the bacterial patterns present in endometriotic lesions, endometrium, and vaginal discharge from women with and without endometriosis, no significant differences were found in the bacteria profiles between control and endometriotic patients.^25^ However, based on another study that aimed at molecular detection of microbial colonization in the cervical mucus of women with and without endometriosis, *Streptococcus* and Enterobacteriaceae in cervical mucus were more frequently detected in women with endometriosis than the women without it.^26^

According to previous studies, the presence of various microorganisms in follicular fluid and secreted cytokines from them may decrease the pregnancy rate and have a negative effect on the IVF/ICSI outcomes, especially in women with endometriosis.^17^, ^27^, ^28^ Based on a study that aimed to investigate the influence of the microbial colonization of follicular fluid on ART outcomes, bacterial colonization in follicular fluid was associated with a decrease in the fertility rate and viable embryos in ART cycles, especially in women with endometriosis.^17^ Contradictorily, based on another study that aimed to investigate the effect of microorganisms present in follicular fluid on IVF outcomes, bacterial colonization of follicular fluid had no effect on the number of retrieved oocytes, fertilization and pregnancy rates, and IVF outcomes.^29^ Based on another research that was performed to identify and compare the microorganisms present in follicular fluid and their influence on IVF/ICSI outcomes, there was no relationship between the microorganisms present in follicular fluid and the embryo quality and the rate of pregnancy in IVF/ICSI cycles.^14^

In the interpretation of the above contradictory findings, it is important to point out that currently the effect of bacteria on the oocyte is not well known and there are relatively few studies have been performed on the microorganisms present in the follicular fluid that might play a potential role in women's fertility.^27^

According to the findings of the present study, the mean number of M II oocytes in women with and without endometriosis was 3.58 ± 4.22 and 4.79 ± 6.51, respectively, and there was a significant statistical difference between the mean numbers of M II oocytes of the two groups of women with and without endometriosis (*p* = 0.034). There was no significant statistical difference between the mean fertilization rate and grades A, B, C, and D embryo qualities of the two groups of women with and without endometriosis.

In line with the results of the present study regarding the mean number of M II oocytes, the fertilization rate, and embryo quality, based on a meta‐analysis study, although endometrioma was associated with a decrease in the number of M II oocytes retrieved in IVF/ICSI cycles, it had no negative influence on the total number of embryos and also on the number of high‐quality embryos.^3^ According to another research that aimed to study the impact of endometrioma cysts on the morphology of oocytes and the fertility outcomes in ICSI cycles, although M II oocytes were fewer in patients with endometrioma, there was no significant statistical difference between the fertility rates of the two groups.^30^ Contrary to the findings of the present research, based on one study that aimed to compare the IVF outcomes in women with and without endometriosis who have decreased ovarian reserves, endometrioma has no influence on the number of retrieved oocytes and the IVF outcomes such as implantation and the rate of live birth.^31^

To interpret the above‐mentioned contradictory findings, it should be noted that endometriosis is an estrogen‐dependent chronic inflammatory disease and its pathogenesis is multifactorial in causing infertility.^1^ The toxic content of endometrioma cysts such as inflammatory mediators, iron, and reactive oxygen species may influence the ovarian and folliculogenesis reserves by inflammation and destruction of the ovarian cortex and result in the qualitative and quantitative decline in oocytes and therefore poor quality of embryos.^10^, ^31^, ^32^ Ovarian reserve is an important factor in evaluating the success rate of ovulation induction and ART.^33^

One of the clinical predictors of inflammation detection is CBC‐based biomarkers. In the present study, this predictor included studying the number of cells and their ratio. Based on the findings of the present research, there was no significant statistical difference between the mean WBC, lymphocytes, neutrophils, NLR, LMR, and PLR in the two groups of women with and without endometrioses. There was no significant statistical difference between the frequency of CRP in the two groups (*p* = 0.999), but there was a significant statistical difference between the mean ESR in the two groups (*p* = 0.018).

In line with the findings of the present study, based on a study that aimed to evaluate the mean platelet volume (MPV), NLR, and PLR in advanced endometriosis with endometrioma, the participants included 94 patients who were divided into three groups that consisted of 33 patients with endometrioma, 28 patients with endometriosis who did not have endometrioma, and 33 patients who underwent laparoscopic tubal ligation. According to the findings of this research, there was no significant statistical difference in the MPV, NLR, and PLR of the three groups.^21^ Based on a study that was performed to evaluate the role of inflammatory markers in detecting extraperitoneal endometriosis, laboratory assessment included the study of CA‐125, thyroid‐stimulating hormone, CBC differential count, MPV, monocyte‐to‐platelet ratio, NLR, LMR, and PLR. In this research, no significant statistical difference was observed in the routine laboratory outcomes of the patients and inflammatory markers. Besides, there was no significant relationship between the inflammatory marker levels in patients who underwent surgery because of endometriosis in different locations with various symptoms.^34^

Contrary to the findings of the present study, based on a study that aimed to investigate the diagnostic value of combination markers of NLR and CA‐125 for differentiating endometrioma from other benign ovarian cysts, the NLR and CA‐125 levels were assessed separately and combined. Based on the findings of this research, neutrophils, lymphocytes, and NLR were higher in women with endometriosis than in women without it. There was also a direct relationship between the stage of the disease and the size of the endometrioma and the rate of NLR increase.^35^

To explain these findings, we may say that although the NLR and PLR in peripheral blood are considered simple markers of systematic inflammatory reactions and disease prognosis and assessing these parameters in the peripheral blood is an available and cost‐effective method,^20^, ^21^, ^34^ more studies are needed in this regard.

## 5 STRENGTHS AND LIMITATIONS

One of the strengths of this study was to evaluate the follicular fluid as an optimal factor in predicting the oocyte quality, fertilization rate, embryo quality, and success rate of ART. Besides, assessing the inflammatory markers of CBC is an available, noninvasive, diagnostic, and cost‐effective method of detecting infection in women with endometriosis and this may provide a valuable context for further future studies. One of the limitations of the present study was that since few studies have been performed on the CBC‐based biomarkers as a clinical predictor of infection diagnosis in women with endometriosis. Therefore, further comparison of the results was not possible. Another limitation of this study is procedural bias. In this study, we attempted to aspirate follicular fluid under completely sterile conditions, but the sample may have been contaminated during aspiration and interfered with the interpretation of the results.

## 6 CONCLUSIONS

There was no significant statistical difference between the frequency of bacteria present in the follicular fluid in the two groups of women with and without endometrioses who were undergoing IVF. There was a significant statistical difference between the mean numbers of oocytes of M II in the two groups. There was no significant statistical relationship between the fertilization rate and grades A, B, C, and D embryo qualities in the two groups. There was no significant statistical difference between the mean WBC, lymphocytes, neutrophils, NLR, LMR, and PLR in the two groups. In our study, there was no statistically significant difference between the frequency of CRP in the two groups, but the mean ESR showed a statistically significant difference. It seems necessary to evaluate follicular fluid as a biological substance that is considered an optimal factor for predicting oocyte quality, fertilization rate, embryo quality, and the success rate of ART.

## AUTHOR CONTRIBUTIONS

**Roya Kabodmehri**: Visualization; writing—original draft; writing—review and editing. **Seyedeh Hajar Sharami**: Conceptualization; data curation; formal analysis; investigation; methodology; writing—original draft; writing—review and editing. **Sedigheh Tavakoli**: Data curation; software; writing—original draft; writing—review and editing. **Yalda Donyaei‐Mobarrez**: Data curation; software; writing—original draft; writing—review and editing. **Ziba Zahiri Sorouri**: Conceptualization; resources; software; supervision; validation; visualization; writing—original draft; writing—review and editing. **Nasrin Ghanami Gashti**: Data curation; software; supervision; writing—original draft; writing—review and editing. **Mohammad hadi Bahadori**: Data curation; formal analysis; supervision; writing—original draft; writing—review and editing. **Forozan Milani:** Data curation; software; validation; visualization; writing—original draft; writing—review and editing.

## CONFLICT OF INTEREST STATEMENT

The authors declare no conflict of interest.

## ETHICS STATEMENT

All methods were performed following the relevant guidelines and regulations. This research is derived from a research project approved with the code IR.GUMS.REC.1400.448 by the Department of Research and Technology and the Ethics Committee of Guilan University of Medical Sciences. Written informed consent was obtained from every participating case after the purpose risk; benefits, confidentiality, and degree of involvement were fully explained to caregivers in their local language before starting the data collection.

## TRANSPARENCY STATEMENT

The lead author Ziba Zahiri Sorouri affirms that this manuscript is an honest, accurate, and transparent account of the study being reported; that no important aspects of the study have been omitted; and that any discrepancies from the study as planned (and, if relevant, registered) have been explained.

## Data Availability

The data sets employed in this study are available with the corresponding author upon reasonable request via email.

## References

[1] Boucher A , Brichant G , Gridelet V , et al. Implantation failure in endometriosis patients: etiopathogenesis. J Clin Med. 2022;11(18):5366. 10.3390/jcm11185366 36143011 PMC9505862

[2] Dong Z , An J , Xie X , Wang Z , Sun P . Preoperative serum anti‐Müllerian hormone level is a potential predictor of ovarian endometrioma severity and postoperative fertility. Eur J Obstet Gynecol Reprod Biol. 2019;240:113‐120. 10.1016/j.ejogrb.2019.06.024 31255930

[3] Alshehre SM , Narice BF , Fenwick MA , Metwally M . The impact of endometrioma on in vitro fertilisation/intra‐cytoplasmic injection IVF/ICSI reproductive outcomes: a systematic review and meta‐analysis. Arch Gynecol Obstet. 2021;303(1):3‐16. 10.1007/s00404-020-05796-9 32979078 PMC7854445

[4] García‐Gómez E , Vázquez‐Martínez ER , Reyes‐Mayoral C , Cruz‐Orozco OP , Camacho‐Arroyo I , Cerbón M . Regulation of inflammation pathways and inflammasome by sex steroid hormones in endometriosis. Front Endocrinol. 2020;10:935. 10.3389/fendo.2019.00935 PMC7000463 32063886

[5] Kimber‐Trojnar Ż , Pilszyk A , Niebrzydowska M , Pilszyk Z , Ruszała M , Leszczyńska‐Gorzelak B . The potential of non‐invasive biomarkers for early diagnosis of asymptomatic patients with endometriosis. J Clin Med. 2021;10(13):2762. 10.3390/jcm10132762 34201813 PMC8268879

[6] Lin W‐C , Chang CY‐Y , Hsu Y‐A , Chiang J‐H , Wan L . Increased risk of endometriosis in patients with lower genital tract infection. Medicine. 2016;95(10):e2773. 10.1097/MD.0000000000002773 26962775 PMC4998856

[7] Bullon P , Navarro JM . Inflammasome as a key pathogenic mechanism in endometriosis. Curr Drug Targets. 2017;18(9):997‐1002. 10.2174/1389450117666160709013850 27397068

[8] Kokot I , Piwowar A , Jędryka M , Sołkiewicz K , Kratz EM . Diagnostic significance of selected serum inflammatory markers in women with advanced endometriosis. Int J Mol Sci. 2021;22(5):2295. 10.3390/ijms22052295 33669013 PMC7956504

[9] Hanege BY , Çekıç SG , Ata B . Endometrioma and ovarian reserve: effects of endometriomata per se and its surgical treatment on the ovarian reserve. Facts Views Vis Obgyn. 2019;11(2):151. 31824636 PMC6897522

[10] Wu Y , Yang R , Lan J , Lin H , Jiao X , Zhang Q . Ovarian endometrioma negatively impacts oocyte quality and quantity but not pregnancy outcomes in women underwent IVF/ICSI treatment: a retrospective cohort study. Front Endocrinol. 2021;12:739228. 10.3389/fendo.2021.739228 PMC8645929 34880831

[11] Zeng C , Lu R , Li X , Kuai Y , Wang Sh , Xue Q . The presence of ovarian endometrioma adversely affect ovarian reserve and response to stimulation but not oocyte quality or IVF/ICSI outcomes: a retrospective cohort study. J Ovarian Res. 2022;15(116):1‐12. 10.1186/s13048-022-01042-9 36273148 PMC9587543

[12] Da Broi MG , Giorgi VSI , Wang F , Keefe DL , Albertini D , Navarro PA . Influence of follicular fluid and cumulus cells on oocyte quality: clinical implications. J Assist Reprod Genet. 2018;35(5):735‐751. 10.1007/s10815-018-1143-3 29497954 PMC5984887

[13] Feng T , Liu Y . Microorganisms in the reproductive system and probiotic's regulatory effects on reproductive health. Comput Struct Biotechnol J. 2022;20:1541‐1553. 10.1016/j.csbj.2022.03.017 35465162 PMC9010680

[14] Kim SM , Won KH , Hong YH , et al. Microbiology of human follicular fluid and the vagina and its impact on in vitro fertilization outcomes. Yonsei Med J. 2022;63(10):941. 10.3349/ymj.2022.0190 36168247 PMC9520042

[15] Najjar AA , Alosaimi EH , Abduljabbar HS , et al. Prevalence of fungi in human follicular fluid and its potential impact on in vitro fertilization process. Arch Pharm Pract. 2020;11(4). https://archivepp.com/article/prevalence-of-fungi-in-human-follicular-fluid-and-its-potential-impact-on-in-vitro-fertilization-process?html

[16] Omar Noor S , Alosaimi E , Najjar A . Detection of human follicular fluid microorganisms at the time of oocyte retrieval and its impact on in‐vitro fertilization outcomes. Prensa Med Argent. 2020;S2:004. 10.47275/0032-745X-S2-004S2-004

[17] Pelzer ES , Allan JA , Cunningham K , et al. Microbial colonization of follicular fluid: alterations in cytokine expression and adverse assisted reproduction technology outcomes. Hum Reprod. 2011;26(7):1799‐1812. 10.1093/humrep/der108 21511711

[18] Pelzer ES , Allan JA , Waterhouse MA , Ross T , Beagley KW , Knox CL . Microorganisms within human follicular fluid: effects on IVF. PLoS One. 2013;8(3):e59062. 10.1371/journal.pone.0059062 23554970 PMC3595219

[19] Diaz‐Martínez MC , Bernabeu A , Lledó B , et al. Impact of the vaginal and endometrial microbiome pattern on assisted reproduction outcomes. J Clin Med. 2021;10(18):4063. 10.3390/jcm10184063 34575174 PMC8465380

[20] Cho S , Cho H , Nam A , et al. Neutrophil‐to‐lymphocyte ratio as an adjunct to CA‐125 for the diagnosis of endometriosis. Fertil Steril. 2008;90(6):2073‐2079. 10.1016/j.fertnstert.2008.03.061 18555226

[21] Yavuzcan A , Caglar M , Ustun Y , et al. Evaluation of mean platelet volume, neutrophil/lymphocyte ratio and platelet/lymphocyte ratio in advanced stage endometriosis with endometrioma. J Turk Ger Gynecol Assoc. 2013;14(4):210‐215. 10.5152/jtgga.2013.55452 24592108 PMC3935542

[22] Khan KN , Fujishita A , Muto H , et al. Levofloxacin or gonadotropin releasing hormone agonist treatment decreases intrauterine microbial colonization in human endometriosis. Eur J Obstet Gynecol Reprod Biol. 2021;264:103‐116. 10.1016/j.ejogrb.2021.07.014 34298448

[23] Jiang I , Yong PJ , Allaire C , Bedaiwy MA . Intricate connections between the microbiota and endometriosis. Int J Mol Sci. 2021;22(11):5644. 10.3390/ijms22115644 34073257 PMC8198999

[24] Lu F , Wei J , Zhong Y , et al. Antibiotic therapy and vaginal microbiota transplantation reduce endometriosis disease progression in female mice via NF‐κB signaling pathway. Front Med. 2022;9:831115. 10.3389/fmed.2022.831115 PMC9005645 35433736

[25] Hernandes C , Silveira P , Rodrigues Sereia AF , et al. Microbiome profile of deep endometriosis patients: comparison of vaginal fluid, endometrium and lesion. Diagnostics. 2020;10(3):163. 10.3390/diagnostics10030163 32192080 PMC7151170

[26] Akiyama K , Nishioka K , Khan KN , et al. Molecular detection of microbial colonization in cervical mucus of women with and without endometriosis. Am J Reprod Immunol. 2019;82(2):1‐9. 10.1111/aji.13147 31087436

[27] Wu YR , Dong YH , Liu CJ , et al. Microbiological composition of follicular fluid in patients undergoing IVF and its association with infertility. Am J Reprod Immunol. 2022;89:e13652. 10.1111/aji.13652 36397134

[28] Vajpeyee M , Yadav LB , Tiwari S , Tank P . To understand the reproductive tract microbiome associated with infertility through metagenomics analysis. Middle East Fertil Soc J. 2021;26(1):31. 10.1186/s43043-021-00078-z

[29] Usman SF , Shuaibu IR , Durojaiye K , Medugu N , Iregbu KC . The presence of microorganisms in follicular fluid and its effect on the outcome of in vitro fertilization‐embryo transfer (IVF‐ET) treatment cycles. PLoS One. 2021;16(2):e0246644. 10.1371/journal.pone.0246644 33556108 PMC7870083

[30] Bilgic BE , Kurek Eken M , Ayla Ş , Kose A , Kutlu T , İlhan G . The rate of oocytes with granular cytoplasm is higher in women with endometrioma in ICSI cycles. J Obstet Gynaecol. 2022;42(3):467‐471. 10.1080/01443615.2021.1916803 34165007

[31] Deng Y , Ou Z , Yin M , Chen Z , Chen S , Sun L . Does current ovarian endometrioma increase the time for DOR patients to reach live birth in IVF? BMC Pregnancy Childbirth. 2022;22(1):324. 10.1186/s12884-022-04670-7 35428243 PMC9011965

[32] Suardi D , Permadi W , Djuwantono T , Hidayat YM , Bayuaji H , Edo Gautama GP . Correlation of serum anti‐Mullerian hormone (AMH) level on ovarian volume in women with endometrioma. Int J Gen Med. 2021;14:1‐8. 10.2147/IJGM.S272071 33442285 PMC7797296

[33] Inal ZO , Engin Ustun Y , Yilmaz N , Aktulay A , Bardakci Y , Gulerman C . Does the anti‐Müllerian hormone truly reflect ovarian response in women with endometrioma? J Obstet Gynaecol. 2019;39(4):516‐521. 10.1080/01443615.2018.1533542 30744464

[34] Gülücü S , Gümüşburun N . The role of inflammatory markers in the diagnosis of extraperitoneal endometriosis. J Exp Clin Med. 2022;39(4):1004‐1007. 10.52142/omujecm.39.4.15

[35] Tokmak A , Yildirim G , Öztaş E , et al. Use of neutrophil‐to‐lymphocyte ratio combined with CA‐125 to distinguish endometriomas from other benign ovarian cysts. Reprod Sci. 2016;23(6):795‐802. 10.1177/1933719115620494 26692541

